# Traditional Chinese Medicine Reduces the Incidence of Chemotherapy-Induced Stroke: A Five-Year Nationwide Population-Based Cohort Study From Taiwan

**DOI:** 10.3389/fphar.2021.614606

**Published:** 2021-05-26

**Authors:** Chien-Chen Huang, Yu-Cih Yang, Iona MacDonald, Ching-Yuan Lai, Cheng-Hao Tu, Yi-Hung Chen

**Affiliations:** ^1^ Graduate Institute of Chinese Medicine, School of Chinese Medicine, College of Chinese Medicine, China Medical University, Taichung, Taiwan; ^2^ Department of Traditional Chinese Medicine, An Nan Hospital, China Medical University, Tainan, Taiwan; ^3^ Management Office for Health Data, China Medical University Hospital, Taichung, Taiwan; ^4^ Graduate Institute of Acupuncture Science, China Medical University, Taichung, Taiwan; ^5^ Center for Emergency and Critical Care Medicine, China Medical University Beigang Hospital, Yunlin, Taiwan; ^6^ Department of Photonics and Communication Engineering, Asia University, Taichung, Taiwan

**Keywords:** traditional Chinese medicine, cancer, chemotherapy, stroke, National Health Insurance Research Database

## Abstract

**Background:** Chemotherapy is suspected to be a risk factor for stroke in patients with cancer, athough the results from large-scale studies are controversial. Few strategies are available for reducing the stroke-related risks.

**Methods:** We analyzed stroke incidence rates in Taiwan’s Longitudinal Health Insurance database 2000 (LHID2000) for patients aged ≥20 years with newly-diagnosed cancer between Jan 1, 2000 and Dec 31, 2006, who did or did not receive chemotherapy. Moreover, we compared stroke incidence rates among chemotherapy users who did or did not use traditional Chinese medicine. All study participants were followed-up for 5 years or until they had a stroke.

**Results:** In adjusted Kaplan-Meier analysis, the incidence of stroke was higher within the first year of cancer diagnosis among chemotherapy recipients compared with those who did not receive chemotherapy (31.1 vs. 9.75; adjusted subdistribution hazard ratio [sHR] 2.21; 95% confidence interval [CI], 1.52–3.20; *p* < 0.001). This between-group difference persisted at 4 years of follow-up (13.6 vs. 5.42; adjusted sHR 1.94; 95% CI, 1.53–2.46; *p* < 0.001). Similarly, the 5-year incidence rate of stroke was significantly lower among chemotherapy recipients using TCM vs. non-TCM users (0.19 vs. 0.46; adjusted sHR 0.45; 95% CI, 0.26–0.79; *p* < 0.001), as was the mortality rate (adjusted sHR 0.55; 95% CI, 0.44–0.68; *p* < 0.001).

**Conclusion:** These Taiwanese data suggest that chemotherapy is a risk factor for stroke and that the use of TCM can significantly mitigate this risk. TCM also appears to reduce the mortality risk associated with chemotherapy.

## Introduction

Stroke affects around 15% of patients with cancer, complicating their physical condition and prognosis (Adams, 2019). A stroke event may occur after the initial diagnosis of cancer (Grisold et al., 2009) or before an underlying cancerous disorder is identified (Taccone et al., 2008). In 1985, a large autopsy study reported that 14.6% of patients with systemic cancer had evidence of cerebrovascular disease (CVD) (Graus et al., 1985). The study researchers found that the usual risk factors for CVD (atherosclerotic infarction and hypertensive hemorrhage) accounted for only a small proportion of the CVDs; most were due to pathophysiological abnormalities relating to the neoplasm such as a direct effect of the tumor, coagulation disorders and thrombocytopenia, infections, and diagnostic or therapeutic procedures (Adams, 2019).

As for evidence linking chemotherapy with thromboembolic events, among 784 patients with non-small cell lung cancer treated with platinum-based chemotherapy, 70% of thromboembolic events occurred during the first two courses of chemotherapy treatment (Mellema et al., 2014). In another cohort of 10,963 patients treated with chemotherapy for various cancers between 1993 and 2004 in a single hospital in Taiwan, 16 ischemic strokes were experienced by 15 patients within 1 month after chemotherapy; the incidence of post-chemotherapy ischemic stroke was 0.137% (15/10,963) and 0.035% (of 16/45,294) chemotherapy cycles were complicated by ischemic stroke (Li et al., 2006). In addition, a large nationwide study from Korea involving 20,707 patients with cancer and 675,594 patients without cancer has reported finding a significantly higher risk of any stroke in patients with cancer than in those without cancer (Jang et al., 2019). In that investigation, chemotherapy was associated with an increased risk of any stroke and also ischemic stroke (Jang et al., 2019). Similarly, Japanese research involving 5,887 patients with cancer treated with chemotherapy and 13,119 patients with cancer who did not receive chemotherapy reported a significantly higher risk of stroke in the chemotherapy group, but this difference was no longer significant after further analyses adjusted for cancer status (Kitano et al., 2020). The study researchers suggested that the apparently elevated risk of stroke associated with chemotherapy was likely due to an advanced cancer stage (Kitano et al., 2020). Thus, whether chemotherapy increases the risk of stroke in patients with cancer is controversial.

As to thromboprophylactic strategy and treatment of venous thromboembolism (VTE) in patients with cancer, routine prophylaxis is not recommended for ambulatory patients with cancer receiving systemic chemotherapy (Lyman et al., 2007; Lyman et al., 2015). Low-molecular-weight heparin (LMWH) is a preferred approach for the initial 5–10 days in cancer patients with established VTE (Lyman et al., 2007; Lyman et al., 2015). For long-term anticoagulant therapy, LMWH is preferred for at least 6 months, and anticoagulation for an indefinite period should be considered (Lyman et al., 2007; Lyman et al., 2015). One review of the use of traditional Chinese medicine (TCM) suggests that TCM provides important supportive roles in cancer treatment by reducing toxicity associated with radiation therapy and chemotherapy, enhancing immunity, improving the clinical efficacy of cancer treatment, and prolonging overall survival (Liu et al., 2015). Other evidence suggests that TCM can regulate oncogenes and tumor suppressor genes, epigenetic modification, the tumor microenvironment, and cancer stem cells (Xiang et al., 2019). However, it is unclear as to whether TCM affects the risk of stroke induced by chemotherapy.

This large-scale, nationwide study investigated whether the incidence of stroke was elevated among cancer patients treated with chemotherapy compared with those who were not. We also examined whether TCM combined with conventional cancer therapy reduced the risk of chemotherapy-induced stroke compared with no TCM use, and we compared mortality rates between TCM users and non-TCM users. Finally, we examined the most commonly used TCM therapies in our study cohort and sought to determine if any were possibly related to a reduced incidence of stroke.

## Methods and Materials

### Study Population and Study Design

This study complied with the Declaration of Helsinki for investigations involving humans and was approved by the Ethics Review Board of Tainan Municipal An-Nan Hospital, China Medical University (TMANH108-REC003). The study was also supported by Taiwan's Ministry of Health and Welfare Clinical Trial Center (MOHW109-TDU-B-212-114004), the MOST Clinical Trial Consortium for Stroke (MOST 109-2321-B-039-002), China Medical University Hospital (DMR-109-231), and the Tseng-Lien Lin Foundation, Taichung, Taiwan. This work was also supported by a grant from China Medical University (CMU109-MF-75) and partially supported by the Chinese Medicine Research Center of China Medical University, under The Featured Areas Research Center Program within the framework of the Higher Education Sprout Project, the Ministry of Education, Taiwan.

This study aimed to assess whether the use of TCM ameliorates the incidence of stroke and mortality in patients with cancer treated with chemotherapy. Thus, we conducted a retrospective cohort study using de-identified claims data between Jan 1, 2000 and Dec 31, 2006 from the Longitudinal Health Insurance Database 2000 (LHID2000), part of Taiwan’s National Health Insurance Research Database (NHIRD) (http://nhird.nhri.org.tw/en/index.htm). We then designed a cohort study using 1:1 frequency matching for the case and control groups.

**FIGURE 1:**
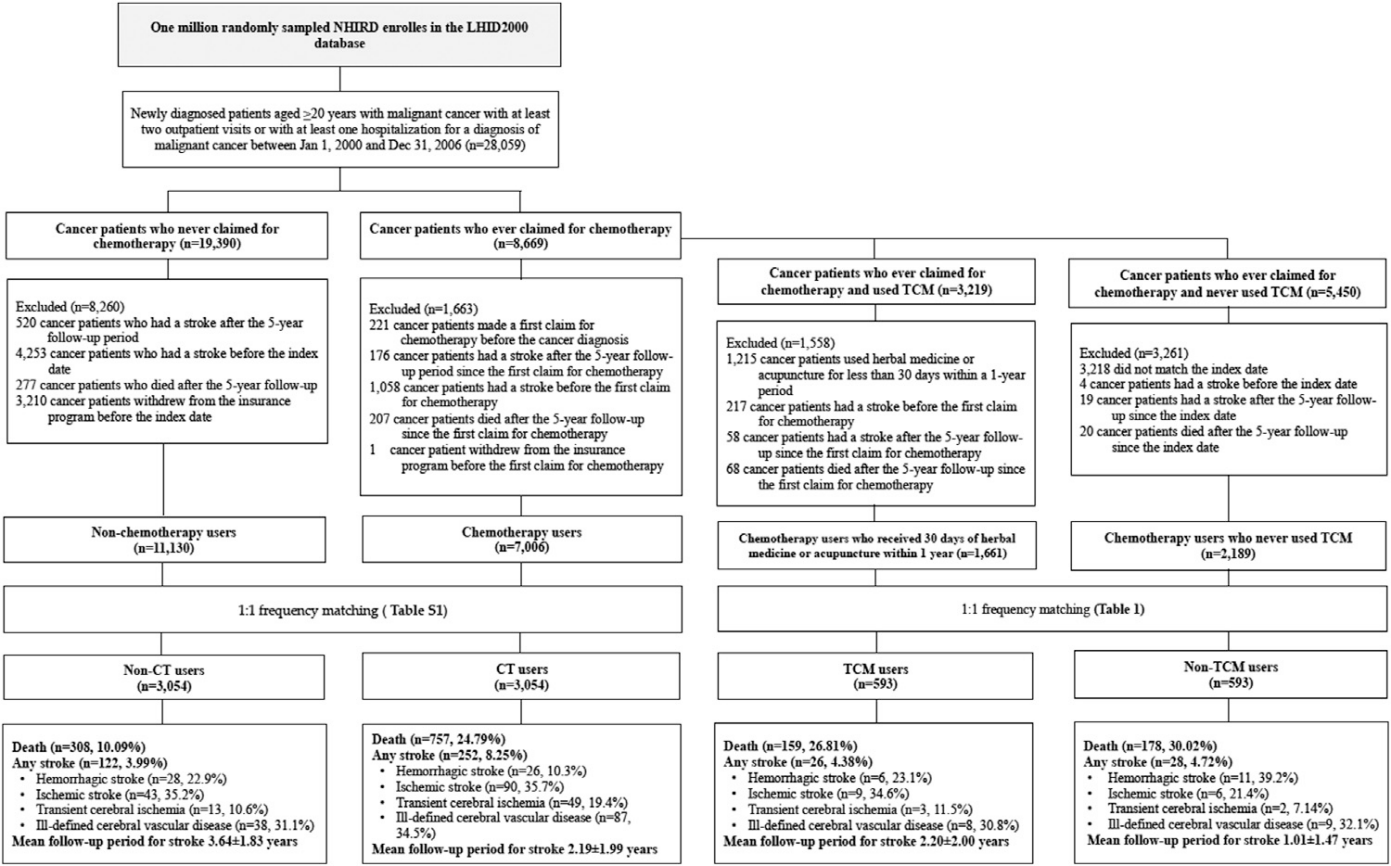
Flowchart of study subject enrolment from the Longitudinal Health Insurance Database 2000 (LHID2000), a sub-dataset of Taiwan's National Health Insurance Research Database (NHIRD), from January 2000 to December 2006. Abbreviations: CT, chemotherapy; TCM, traditional Chinese medicine.

We obtained data for 28,059 patients aged ≥20 years with newly diagnosed malignant cancer (International Classification of Diseases, Ninth Revision, Clinical Modification [ICD-9-CM] codes 140–209) who made at least two outpatient clinic visits or who had at least one hospitalization for malignant cancer between Jan 1, 2000 and Dec 31, 2006, then followed each subject for 5 years (Figure 1). We obtained clinicodemographic data for 8,669 patients treated with chemotherapy in the first year of their cancer diagnosis and for 19,390 patients who never claimed for chemotherapy after their cancer diagnosis. We excluded patients who received chemotherapy before a cancer diagnosis, patients with stroke prior to chemotherapy, and those with stroke diagnosed 5 years after the first chemotherapy administration. We also excluded those who withdrew from the insurance program before the first chemotherapy administration. This resulted in 7,006 cancer patients (cases). The date of the first chemotherapy administration after the diagnosis of cancer was defined as the index date. We then randomly selected the index date among cancer patients who never claimed for chemotherapy and after applying the same exclusion criteria that were used for the cases, we selected 11,130 controls. A process of 1:1 frequency matching by age, gender, the year of first receipt of chemotherapy treatment, cancer treatment-related surgery and radiotherapy, non-chemotherapy medications (targeted therapy, immunotherapy, and hormonal therapy) and diseases related to stroke resulted in 3,054 cases defined as chemotherapy users and 3,054 controls defined as non-chemotherapy users. These 7,108 patients were followed-up for 5 years from the index year or until they had a stroke.

We then investigated the effects of TCM combined with conventional cancer therapy on the risk of stroke. For our study, TCM included Chinese herbal medicine (CHM) and acupuncture treatment. We only included TCM treatment that was supported by more than 30 days of CHM prescription or 30 sessions of acupuncture therapy within a 1-year period. Of 8,669 cancer patients who first received chemotherapy, 3,219 patients had ever used TCM and 5,450 had never previously used TCM. So that our observations were limited to those patients administered TCM who had a stroke within 5 years of first chemotherapy treatment, we excluded patients who received chemotherapy before the cancer diagnosis, patients with stroke before chemotherapy and those with stroke 5 years after their first chemotherapy treatment. We also excluded patients who withdrew from the insurance program before the first chemotherapy administration. After excluding cancer patients who died more than 5 years after the first receipt of chemotherapy, we had 1,661 cancer patients who received chemotherapy and TCM and 2,189 controls who never used TCM. After applying 1:1 frequency matching by age, gender, the year of first receipt of chemotherapy treatment, cancer-related surgery and radiotherapy, non-chemotherapy medications (targeted therapy, immunotherapy, and hormonal therapy), and diseases related to stroke, each cohort contained 593 patients. All patients were followed-up for 5 years after the first year of chemotherapy treatment or until they had a stroke.

### Measurement Outcomes, Comorbidities, and Medication Use

The primary outcome of this study was to compare the incidence of stroke between chemotherapy users and TCM users; the secondary outcome was to evaluate the risk of mortality among chemotherapy recipients using TCM compared with non-TCM users.

Stroke is a disabling disease induced by a multitude of factors (Meschia et al., 2014; Diener & Hankey, 2020). The risk factors for stroke in cancer patients are also complex (Adams, 2019; Dardiotis et al., 2019). Based on our clinical expertize, literature review (Adams., 2019; Dardiotis et al., 2019), and available data in the LHID2000, we selected variables associated with the study outcome (stroke event) that were paired in the case and control groups. We also selected cancer-related treatments that included surgery, radiotherapy, and cancer-related medications, such as immunotherapies, targeted therapies, and hormonal therapies. These treatments are risk factors for stroke in cancer patients. Our analysis also considered comorbidities that cause a stroke. According to previous studies (Lai et al., 2018; Jang et al., 2019), we selected the following comorbidities related to stroke: hyperlipidemia (ICD-9-CM code 272); diabetes mellitus (ICD-9-CM code 250); hypertensive disease (ICD-9-CM codes 401–405); coronary heart disease (ICD-9-CM codes 410–414); acute pulmonary heart disease (ICD-9-CM code 415); cardiac dysrhythmia (ICD-9-CM code 427); congestive heart failure (ICD-9-CM code 428); atherosclerosis (ICD-9-CM code 440); peripheral vascular disease (ICD-9-CM code 443.9); and other venous embolism and thrombosis (ICD-9-CM code 453). Patients with stroke were identified using ICD-9-CM codes 430 to 438.

Records of cancer-related surgery and radiotherapy, as well as cancer-related medications, were all obtained from the Bureau of National Health Insurance. Details of the types of chemotherapies, immunotherapies, targeted therapies and hormonal therapies were obtained from the IBM Micromedex® database. Our analysis included 852 chemotherapeutic agents (anthracyclines, antimetabolites, alkylating agents, mitotic inhibitors, topoisomerase inhibitors, and proteasome inhibitors), 57 immunotherapy agents (immune suppressants, antiproliferative agents, pyrimidine nucleoside analogs, and interleukin), 135 targeted therapy agents (tyrosine kinase inhibitors, antibody drug conjugates, monoclonal antibodies, immunological agents, and BRAF inhibitors) and 123 hormonal agents (antiandrogen agents, aromatase inhibitors, endocrine/metabolic agents, antiestrogen agents, gonadotropin-releasing hormone agonists, and adrenocortical suppressants).

### Statistical Analysis

Categorical variables are expressed as total numbers and percentages. Continuous variables are presented as means ± standard deviations (SDs). For comparisons between cases (chemotherapy and TCM users) and controls (non-chemotherapy and non-TCM users), the Chi-square test was performed for categorical variables and the two-sample *t*-test for continuous variables. Time-to-event outcomes were analyzed using the Kaplan-Meier method and multivariable Cox proportional hazards regression. Covariates included in the Cox models for the entire cohort were age, gender, cancer-related treatments (i.e., surgery, radiotherapy, immunotherapy, targeted therapy, and hormonal therapy) and comorbidities in stroke (i.e., hyperlipidemia, diabetes mellitus, hypertensive disease, coronary heart disease, acute pulmonary heart disease, cardiac dysrhythmia, congestive heart failure, atherosclerosis, peripheral vascular disease, other venous embolism and thrombosis), which were included in the adjusted analyses of the case and control cohorts.

We used SAS statistical software version 9.4 for Windows (SAS Institute Inc., Cary, NC, United States) and Stata-14 software (StataCorp, College Station, TX, United States). Cox proportional hazard modeling calculated the adjusted hazard ratios (aHRs) and 95% confidence intervals (CIs) for the cumulative risk of death. The Fine and Gray model was used to estimate the subdistribution hazard ratios (sHRs) of stroke by considering death as a competing risk (Fine & Gray, 1999). The Aalen-Johansen estimator (Gooley et al., 1999; Kim, 2007) compared Kaplan-Meier analyses with competing risk cumulative incidence curves. Multivariate models adjusted for age, gender, comorbidities, and treatment of cancer, aspirin use, coagulopathy and disseminated intravascular coagulopathy. Log-rank testing estimated differences in survival between cases and controls. We used the Hosmer and Lemeshow goodness-of-fit test to verify the fitness of the multivariate model in this study. The resulting *p*-value of 0.77 > 0.05 indicates a good fit. To address the concern of constant proportionality, we tested for scaled Schoenfeld residuals in the proportional hazard model to evaluate how use of CT and TCM affect the risk of stroke. A two-sided *p*-value of <0.05 was considered statistically significant.

## Results

Table 1 and Supplementary Table S1 describe the demographic and clinical details for all study participants. Of 28,059 patients newly diagnosed with cancer, 8,669 (30.9%) patients ever used chemotherapy after their cancer diagnosis. In this study, chemotherapy users were more likely to be male than female (59.5 vs. 40.5%). The majority of chemotherapy users were aged over 50 years (50–59 years: 22.3%; 60–69 years: 22.6%; ≥70 years: 26.4%); mean age did not differ significantly from non-chemotherapy users (*p* = 0.79). Of the 3,054 chemotherapy users, 31.6% underwent cancer-related surgery, 12.7% received radiotherapy, 0.39% were given targeted therapy, 2.82% received hormonal therapy and none received immunotherapy; use of these therapies did not differ significantly between chemotherapy users and non-chemotherapy users (*p* > 0.99) (Supplementary Table S1). The most frequent comorbidities among chemotherapy users were hyperlipidemia (2.95%), diabetes mellitus (3.93%), hypertensive disease (2.88), and coronary heart disease (1.34%). Between-group differences in comorbidities for chemotherapy users and non-chemotherapy users were not significant (*p* > 0.99). The mean follow-up for stroke was significantly shorter for chemotherapy users than for non-chemotherapy users (2.19 ± 1.99 years vs. 3.64 ± 1.83 years; *p* < 0.0001). The locations of cancers in CT users and non-CT users are shown in Supplementary Table S2.

**TABLE 1:** Baseline characteristics for TCM users and non-TCM users (controls).

	TCM users (*n* = 593)	Non-TCM users (*n* = 593)	*p*-value
Gender (*n*, %)			>0.99^a^
Female	205 (34.6)	205 (34.6)	
Male	388 (65.4)	388 (65.4)	
Age, years (*n*, %)			0.92^a^
20–29	14 (2.36)	10 (1.69)	
30–39	29 (4.89)	34 (5.73)	
40–49	125 (21.1)	126 (21.2)	
50–59	157 (26.5)	148 (24.9)	
60–69	147 (24.8)	150 (25.3)	
≥70	121 (20.4)	125 (21.1)	
Mean (SD)	57.8 (12.9)	58.0 (12.9)	0.79^b^
Cancer-related therapy (n, %)			
Surgery	99 (16.7)	99 (16.7)	>0.99^a^
Radiotherapy	134 (22.6)	134 (22.6)	>0.99^a^
Medication (n, %)			
Immunotherapy	0 (0)	0 (0)	--
Targeted therapy	8 (1.35)	8 (1.35)	>0.99^a^
Hormonal therapy	14 (2.36)	14 (2.36)	>0.99^a^
Stroke-related comorbidities (n, %)			
Hyperlipidemia	1 (0.17)	1 (0.17)	>0.99^a^
Diabetes mellitus	1 (0.17)	1 (0.17)	>0.99^a^
Hypertensive disease	6 (1.01)	6 (1.01)	>0.99^a^
Coronary heart disease	1 (0.17)	1 (0.17)	>0.99^a^
Acute pulmonary heart disease	0 (0)	0 (0)	--
Cardiac dysrhythmia	1 (0.17)	1 (0.17)	>0.99^a^
Congestive heart failure	4 (0.67)	4 (0.67)	>0.99^a^
Atherosclerosis	0 (0)	0 (0)	--
Peripheral vascular disease	0 (0)	0 (0)	--
Other venous embolism and thrombosis	0 (0)	0 (0)	--
Mean (SD) follow-up for stroke (years)	2.20 (2.00)	1.01 (1.47)	<0.0001^b^
Mean (SD) follow-up for death (years)	2.26 (2.02)	1.08 (1.51)	<0.0001^b^

Abbreviations: TCM, traditional Chinese medicine; SD, standard deviation.

Unable to calculate because there were either too few or no events.

a Chi-square test.

b Two-sample *t*-test.

TCM users were predominantly male (male: 65.4%; female: 34.6%). TCM use was much more common among patients aged ≥40 years than younger-aged patients. Mean age did not differ significantly between the TCM and non-TCM groups (*p* = 0.79) (Table 1). All TCM users received chemotherapy, 16.7% underwent surgery, 22.6% received radiotherapy, 1.35% received targeted therapy, 2.36% received hormonal therapy, and none received immunotherapy. Between-group differences for cancer-related treatments among TCM users and non-TCM users were not significant (Table 1). Comorbidities for stroke did not differ significantly between TCM users and non-TCM users; the most common comorbidities among TCM users were hypertensive disease (1.01%) and congestive heart failure (0.67%). The mean follow-up period of stroke was significantly prolonged for TCM users compared with non-TCM users (2.20 ± 2.00 years vs. 1.01 ± 1.47 years; *p* < 0.0001) (Table 1). The locations of cancers in TCM users and non-TCM users are shown in Supplementary Table S3.

### Higher Incidence Rates of Stroke Among Chemotherapy Users Than Among Non-chemotherapy Users

**FIGURE 2:**
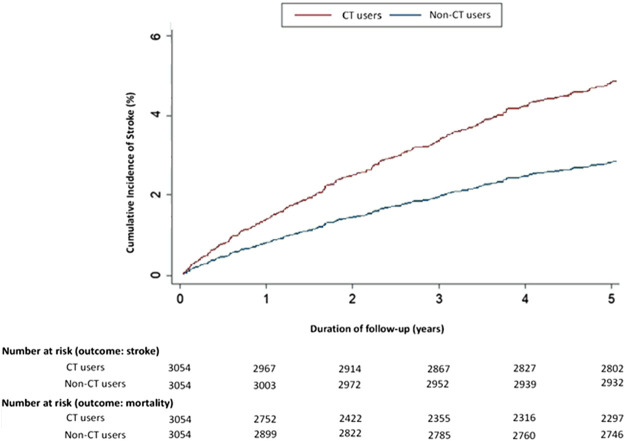
Cumulative incidence rates of stroke in 2000–2006 differed significantly in adjusted Kaplan-Meier estimates for cancer patients administered chemotherapy and those who were not. Abbreviations: CT, chemotherapy.

While testing for scaled Schoenfeld residuals in the proportional hazard model to evaluate how use of CT affects the risk of stroke, no significant relationship was observed between Schoenfeld residuals for CT and follow-up time (*p*-value 0.7512; *p* > 0.05). In adjusted Kaplan-Meier curves, the cumulative incidence of stroke was consistently higher among chemotherapy users than among non-chemotherapy users (Year 1: chemotherapy users 1.5% vs. non-chemotherapy users 1%; Year 5: chemotherapy users 5% vs. non-chemotherapy users 2.5%) (Figure 2). Incidence rates (IRs) and sHRs for stroke were significantly higher among chemotherapy users than among non-chemotherapy users, especially within the first year (chemotherapy users IR 31.1 vs. non-chemotherapy users IR 9.75; adjusted sHR 2.21; 95% CI, 1.52–3.20; *p* < 0.0001) and sustained through year 4 (Year 4: chemotherapy users IR 13.6 vs. non-chemotherapy users IR 5.42; adjusted sHR 1.94; 95% CI, 1.53-2.46; *p* < 0.001) (Supplementary Table S4).

### TCM Reduced the Incidence Rate of Stroke, Especially Ischemic Stroke

**FIGURE 3:**
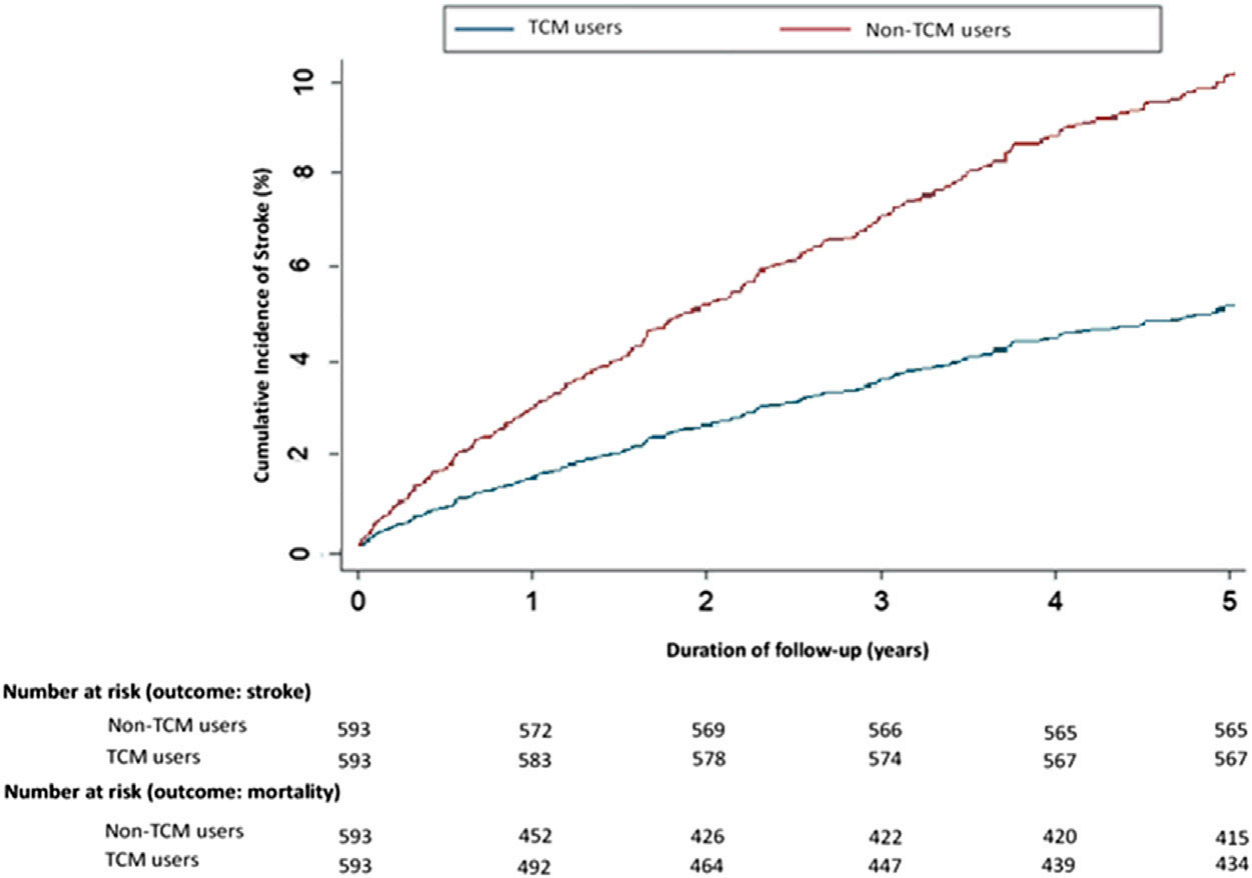
Cumulative incidence rates of stroke in 2000–2006 differed significantly in adjusted Kaplan-Meier estimates for chemotherapy recipients who received TCM for more than 30 days within 1 year and those who did not. Abbreviations: TCM, traditional Chinese medicine.

When we evaluated how the use of TCM affects the risk of stroke throughout the 5-year follow-up period, we found a significant relationship between Schoenfeld residuals for TCM and follow-up time, suggesting the proportionality assumption was violated (*p*-value 0.0154; *p* < 0.05). In subsequent analyses, we stratified the follow-up duration to deal with the proportional hazard assumption violation, as revealed in Supplementary Table S5. Kaplan-Meier analysis with competing risk cumulative incidence curves revealed a lower cumulative incidence of stroke among TCM users compared with non-TCM users (Year 5: TCM users 10% vs. non-TCM users 5%) (Figure 3). The IRs of stroke at 5 years were 0.19 for the TCM cohort and 0.46 for the non-TCM cohort (Supplementary Table S6). TCM users had a significantly lower adjusted sHR of stroke compared with non-TCM users at the end of 5 years of follow-up (1,306 per 10 PY vs. 604 per 10 PY; adjusted sHR 0.45; 95% CI, 0.26–0.79; *p* < 0.001) (Supplementary Table S6). Ischemic stroke was more common than hemorrhagic stroke among TCM users (34.6 vs. 23.1% of patients); TCM users were at significantly lower risk of ischemic stroke than non-TCM users (adjusted sHR 0.11; 95% CI, 0.02–0.62; *p* < 0.05) (Supplementary Table S7).

### CHM Use Amongst Chemotherapy Users Without Incidence of Stroke

Among 593 TCM users, 567 patients had no stroke and 26 patients had a stroke during the 5-year follow-up. Of the TCM users without stroke (*n* = 567), 560 patients were prescribed more than 30 days of CHM, and seven patients received more than 30 sessions of acupuncture only. Of the 560 patients using CHM, 73 also received more than 30 acupuncture sessions. Of the 26 patients with stroke who received CHM, six also received more than 30 acupuncture sessions at the same time. Supplementary Tables S8 and S9 list the average daily doses, average durations of prescriptions, and effects/usage of the 10 most commonly prescribed Chinese formulas and single herbs for cancer patients using CT with or without stroke (Chen, 2019). The top three commonly used CHM formulas were (from most to least) Xiang-Sha-Liu-Jun-Zi-Tang (XSLJZT), Ban-Xia-Xie-Xin-Tang (BXXXT), and Xiao-Chai-Hu-Tang (XCHT) in the non-stroke cohort (Supplementary Table S8). The most commonly used single herbs were Dan Shen (*Salvia miltiorrhiza Bunge*), Bai Hua She She Cao (*Scleromitrion diffusum (Willd.) R. J. Wang*), and Da Huang (*Rheum palmatum L.; Rheum tanguticum Maxim. ex Balf.; Rheum officinale Baill.*) in the non-stroke cohort (Supplementary Table S9). To identify those beneficial CHMs that potentially reduce the risk of ischemic stroke, we excluded duplicates in the top 10 CHM formulas and single herbs in TCM users with and without stroke (Supplementary Tables S8 and S9). CHMs that were screened out from TCM users without stroke and considered possibly beneficial for reducing the risk of ischemic stroke are listed in Table 2. Then, we excluded duplicate CHMs in TCM users with stroke (Supplementary Tables S8 and S9), and screened for CHMs that are possibly not beneficial for ischemic stroke from TCM users with stroke (Table 3). A significant correlation between the daily CHM dosage and the risk of stroke was observed in Table 4.

**TABLE 2:** Potentially beneficial CHMs for ischemic stroke in cancer patients who used chemotherapy between Jan 1, 2000 and Dec 31, 2006, at 5 years of follow-up.

Chinese herbal medicine name	Numbers of prescriptions	Average daily doses (g)	Average duration of prescription (days)	Effects (Chen, 2019)
Gan-Lu-Yin (GLY)	226	12.2	7.7	Nourish yin and clear dampness-heat
Bu-Zhong-Yi-Qi-Tang (BZYQT)	216	4.4	10.9	Tonify the middle and replenish qi, harmonize and tonify the spleen and stomach
Suan-Zao-Ren-Tang (SZRT)	213	8.7	7.8	Nourish blood and tranquilize the mind, clear heat and relax the mind
Shao-Yao-Gan-Cao-Tang (SYGCT)	210	17.8	7.5	Relax tesion to relieve pain
Jia-Wei-Xiao-Yao-San (JWXYS)	176	8.5	9.6	Soothe the liver and release depression, clear heat to cool the blood
Shu-Jing-Huo-Xie-Tang (SJHXT)	174	29.7	6.9	Soothe menstruation, activate blood and dispel wind
Ma-Zi-Ren-Wan (MZRW)	173	2.8	7.8	Nourish the intestine and discharge heat, promote circulation of Qi and relax the bowels
Dan Shen *(Salvia miltiorrhiza Bunge)*	478	2.5	10.6	Blood-activating and stasis-dispelling medicinal
Da Huang *(Rheum palmatum L.) (Rheum tanguticum Maxim. ex Balf.) (Rheum officinale Baill.)*	346	0.9	8.7	Offensive purgative medicinal
Huang Qin *(Scutellaria baicalensis Georgi)*	225	5.6	7.9	Heat-clearing and dampness-drying medicinal
Gan Cao *(Glycyrrhiza uralensis Fisch.) (Glycyrrhiza glabra L.) (Glycyrrhiza inflata Batalin)*	219	1.4	7.7	Qi-tonifying medicinal
Gua Lou Gen *(Trichosanthes rosthornii Harms)*	195	1.1	7.7	Heat-clearing and fire-purging medicinal
Mai Men Dong *(Ophiopogon japonicus (Thunb.) Ker-Gawl)*	195	2.0	8.7	Yin-tonifying medicinal

**TABLE 3:** CHMs that are probably not beneficial for ischemic stroke in cancer patients who used chemotherapy between Jan 1, 2000 and Dec 31, 2006, at 5 years of follow-up.

Chinese herbal medicine name	Numbers of prescriptions		Average daily doses (g)		Average duration of prescription (days)	Effects (Chen, 2019)
Ping-Wei-San (PWS)	37		48.3		5.9	Dry dampness to fortify the spleen, regulate qi and harmonize the middle
Jing-Fang-Bai-Du-San (JFBDS)	30		5.4		6.1	Promote sweating to release the exterior, disperse wind and dispel dampness
Qing-Xin-Li-Ge-Tang (QXLGT)	28		5.5		6	Clear heat and detoxicate
Huo-Xiang-Zheng-Qi-San (HXZQS)	25		4.5		6.2	Release the exterior and resolve dampness, regulate qi and harmonize the middle
Ma-Xing-Gan-Shi-Tang (MXGST)	24		3		6	Diffusion with pungent-cool, clear the lung to calm panting
Zhi-Bo-Di-Huang-Wan (ZBDHW)	22		3.3		6.8	Nourish yin to downbear fire
Tiao-Wei-Cheng-Qi-Tang (TWCQT)	21		3.9		6.1	Soften hardness to relax the bowels, harmonize the stomach and discharge heat
Hai Piao Siao *(Sepiella maindroni) (Sepia esculenta)*	27		1		5.5	Astringent medicinal
Jie Geng *(Platycodon grandiflorus (Jacq.) A.DC.)*	21		1.2		6.6	Heat-phlegm clearing and resolving medicinal
Yin Chen *(artemisia capillaris Thunb.)*	18		1.1		7	Dampness-draining diuretic medicinal
Bai Ji *(Bletilla striata (Thunb.) Rchb.f.)*	17		1.1		6.2	Hemostatic medicinal
Suan Zao Ren *(Ziziphus jujuba Mill.)*	17		1.1		6.8	Heart-nourishing tranquilizing medicinal
Fu Shen *(Poria cocos (Schwein.) F.A.Wolf)*	16		1.1		6.8	Heart-nourishing tranquilizing medicinal

**TABLE 4:** Hazard ratios and confidence intervals of stroke risk associated with the average daily CHM dosage.

Daily CHM dosage	Numbers of Chinese herb users	Numbers of patients with stroke	Crude HR(95% CI)	Adjusted HR^a^(95% CI)
Less than 15.9 (g)	201	11	1 (reference)	1 (reference)
15.9–19.7 (g)	214	8	0.77 (0.68–0.97)^b^	0.71 (0.65–0.83)^b^
More than 19.7 (g)	178	7	0.64 (0.61–0.88)^b^	0.61 (0.58–0.79)^c^

Abbreviations: CHM, Chinese herbal medicine; CI, confidence interval.

a The model was adjusted by gender, age, index year, cancer-related therapy and stroke-related comorbidities, aspirin use, coagulopathy and disseminated intravascular coagulopathy.

b *p* < 0.05.

c *p* < 0.01.

### TCM Lowered Mortality Rates in the Chemotherapy Cohort

**FIGURE 4:**
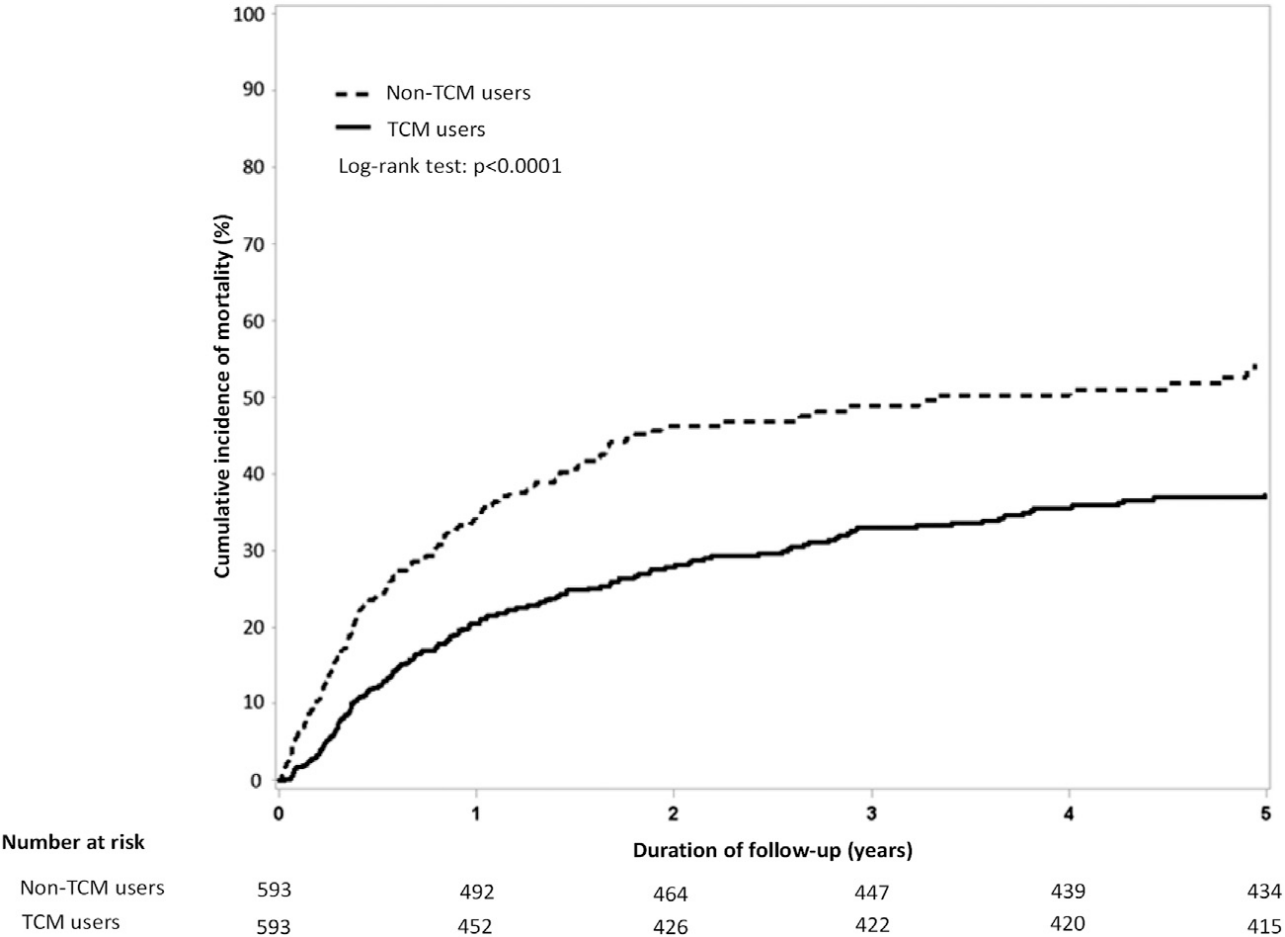
Comparison of cumulative incidence of mortality among chemotherapy users who did and did not use TCM. Abbreviations: TCM, traditional Chinese medicine.

By the end of the 5-year follow-up, Kaplan-Meier analysis revealed fewer deaths among chemotherapy users who received TCM vs. those who did not receive TCM (159 vs. 178; Log-rank test, *p* < 0.0001; Figure 4 and Supplementary Table S10). The IR of death was lower in TCM users compared with non-TCM users from the end of Year 2 (6.14) and persisted to the end of Year 5 (1.18) (Supplementary Table S10). The aHR was lower in TCM users compared with non-TCM users from the end of Year 2 (0.78; 95% CI, 0.61–0.98; *p* = 0.03) and persisted to the end of Year 5 (0.55; 95% CI, 0.44–0.68; *p* < 0.0001) (Supplementary Table S10).

## Discussion

In this study, around one-third (30.9%) of cancer patients received chemotherapy and these patients were at higher risk of all-cause stroke than their counterparts who did not receive chemotherapy; notably, the risk of stroke was higher within the first year of follow-up than at any other time. Similar results have been described in two studies, the first of which detailed how over a 2-year follow-up period, the majority of thromboembolic events occurred in the first two cycles in patients with non-small cell lung cancer during treatment with platinum-based chemotherapy (Mellema et al., 2014). In the second study, which discusses 11 years of follow-up for 10,963 patients from a single hospital cancer database, 75% of ischemic strokes occurred within 10 days of the last chemotherapy administration; 62.5% occurred after the first cycle of chemotherapy (Li et al., 2006). Our data suggest that after adjusting for cancer-related therapy and potential confounding factors, the risks of stroke and mortality were lower for cancer patients administered chemotherapy plus TCM compared with those patients who received chemotherapy only. To the best of our knowledge, this is the first large-scale, nationwide cohort study to assess the effects of TCM treatment in cancer patients receiving chemotherapy.

The de-identified data from the LHID2000 included each study participant’s gender, date of birth, clinical visits, hospitalizations, medical prescriptions and doses, and treatment procedures. Misclassification bias concerning the ICD-9-CM codes is unlikely, because the diagnoses for stroke and cancer were all recorded by physicians. All chemotherapy treatments analyzed in this study were conventional, nontargeted therapies. All details of prescribed intervals and doses of dosing regimens (including chemotherapies and TCMs) are recorded in Taiwan's NHIRD, as well as in the records from individual hospitals, clinics and healthcare facilities.

Although chemotherapy is clearly a critical risk factor for stroke (Falanga, 1998; Adams, 2019), the evidence for a relationship between thromboembolic events and chemotherapy is controversial (Jang et al., 2019; Kitano et al., 2020). As mentioned earlier, in two long-term investigations into the association between chemotherapy and the risk of stroke in patients with cancer, one found that chemotherapy increased the risk of any stroke and ischemic stroke in adjusted analyses that included 7 years of data (Jang et al., 2019), while the other study included 8 years of follow-up data and reported a significantly higher risk for stroke in the chemotherapy cohort compared with the non-chemotherapy cohort (Kitano et al., 2020). However, after adjusting for cancer status using inverse probability of treatment weight-adjusted analysis and stratified Cox regression modeling, this between-group difference was no longer significant, so the study researchers suggested that the advanced cancer stage, rather than chemotherapy, increased the risk of stroke (Kitano et al., 2020). In the present study, the cumulative incidence of stroke increased each year during the 5-year study period among chemotherapy users and the IRs and sHRs for stroke were consistently higher among chemotherapy users compared with non-chemotherapy users. The findings indicate that cancer-related chemotherapy is a risk factor for stroke.

As to the mechanisms underlying the chemotherapy-related stroke risk, abundant evidence attests to an association between chemotherapy and cerebral thrombotic events (Saynak et al., 2008; Tully et al., 2016; Kanjanapan et al., 2020). Four mechanisms may contribute to chemotherapy-related thrombosis (Falanga, 1998): the release of procoagulants and cytokines from chemotherapy-damaged cancer cells; chemoradiotherapy-induced damage of the vascular endothelium can result in pulmonary thrombosis and impair the permeability of glomerular cells, leading to a nephrotic syndrome, hypercoagulation and a higher likelihood of thrombosis; elevated tissue factor procoagulant activity, reflected by higher levels of monocyte and macrophage expression; and reductions in plasma levels of naturally occurring anticoagulants (antithrombin, protein C and protein S).

TCM is accepted in many countries for stroke patients (Yeh et al., 2017; Liu et al., 2018). In Taiwan, TCM is very frequently prescribed for stroke patients (Liao et al., 2012), with over half of all stroke patients attending TCM outpatient clinics between 2001 and 2009 receiving both CHM and acupuncture/traumatology treatment (Chang et al., 2016). In Taiwanese studies, TCM treatment reduced mortality in stroke (Chang et al., 2016) and adult cancer patients (Kuo et al., 2018). In line with previous studies, our study shows that cancer treatments, such as chemotherapy, increase the incidence of stroke. It is important to understand whether TCM affects the incidence of stroke among cancer patients receiving chemotherapy.

We believe that our evidence is the first to demonstrate a reduced risk of stroke among cancer patients receiving TCM (CHM prescriptions and/or acupuncture) with chemotherapy. In particular, the risk of ischemic stroke was significantly lower among TCM users compared with non-TCM users. Based on the results of Shih’s study (Shih et al., 2015), the beneficial effects of acupuncture may not be restricted to improvements in physical activity. It has been shown that acupuncture is useful in lowering blood pressure (Flachskampf et al., 2007; Kim et al., 2006), reducing the expression of inflammatory mediators (Choi et al., 2010) and improving the lipid profile (Cabioglu & Ergene, 2005; Cabioglu et al., 2008). Receiving acupuncture treatment in combination with medications for stroke prevention appears to lower the stroke recurrence rate by a greater extent compared with either treatment modality alone (Shih et al., 2015). Among the CHMs in Table 2, Shu-Jing-Huo-Xie-Tang (SJHXT), Ma-Zi-Ren-Wan (MZRW) and Dan Shen (*Salvia miltiorrhiza* Bunge) are some of the most frequently prescribed prescriptions for subjects with ischemic stroke by TCM doctors in Taiwan (Hung et al., 2015). When used with warfarin, a higher dose of the CHM formula Shu-Jing-Hwo-Shiee-Tang (SJHST, an alternative name to SJHXT), can enhance the anticoagulant effects of warfarin, whereas SJHST alone has shown no anticoagulation effect (Yang et al., 2013). Furthermore, SJHST may inhibit intrinsic coagulation pathways, which needs further investigation (Yang et al., 2013). MZRW is the most commonly prescribed herbal formula for subjects with constipation in Taiwan (Hung et al., 2015). MZRW increases spontaneous bowel movements and relieves the severity of constipation and straining during evacuation (Hung et al., 2015). The relationship between MZRW and stroke prevention remains unclear. The pharmacological effects of Dan Shen have been widely evaluated and include dilation of the cardiocerebral vessels, improvements in cerebral microcirculation, inhibition of post-ischemic cerebral β-endorphine elevations, inhibition of coagulation and activating thrombolysis, suppression of platelet aggregation, scavenging of free radicals to prevent ischemic reperfusion injury, reductions in cerebral edema, removal of blood stasis, and enhanced tolerance to hypoxia in ischemic tissue (Hung et al., 2016). The active ingredients in Dan Shen have antioxidative, antiatherosclerotic and anti-inflammatory effects that contribute to neuroprotective effects (Hung et al., 2015). Furthermore, Da Huang (*Rheum palmatum L./Rheum tanguticum Maxim./Rheum officinale Baill.*) shows anti-inflammatory and antioxidative activity, and decreases the incidence of brain injury after ischemic stroke (Hung et al., 2016). In addition, components of Bu-Zhong-Yi-Qi-Tang (BZYQT), including Huang Qi (*Astragalus membranaceus* (Fisch.) Bunge), Dang gui (*Angelica sinensis* (Oliv.) Diels), Bai Jhu (*Atractylodes macrocephala* Koidz.) and Gan Cao (*Glycyrrhiza uralensis* Fisch.) have shown neuroprotective effects in ischemic stroke. Huang Qi isolates calycosin and formononetin have shown neuroprotective effects in cerebral ischemic rats (Ip et al., 2016), while ferulic acid and ligustilide isolated from Dang gui have shown neuroprotection in rodent cells and ischemic rats (Ip et al., 2016). Liquiritin, a major constituent of Gan Cao, enhances the neurotrophic effects of nerve growth factor and protects against Aβ-induced toxicity in rat cortical neurons and ischemic mice (Ip et al., 2016). The antioxidative effects of Bai Jhu result from its active ingredients, the flavonoids and phenolic acids, which are associated with metal-chelating and radical-scavenging activity (Li et al., 2012). Instead, most CHMs listed in Table 3 are used to treat upper respiratory infections, such as Jing-Fang-Bai-Du-San (JFBDS), Qing-Xin-Li-Ge-Tang (QXLGT), Ma-Xing-Gan-Shi-Tang (MXGST), Jie Geng (*Platycodon grandiflorus* (Jacq.) A.DC.) and do not exhibit any benefit for stroke. Our analysis suggests that TCM could be an appropriate and safe therapeutic option for long-term stroke prophylaxis in patients with cancer receiving chemotherapy.

As shown by previous research, mortality is increased in cancer patients with stroke (Jang et al., 2019) and chemotherapy increases the risk of death in patients with cancer (adjusted cause-specific HR 2.91; 95% CI, 2.74–3.08) (Jang et al., 2019). In a cohort of 784 patients with non-small cell lung cancer treated with platinum-based chemotherapy, 63 (8.0%) experienced thromboembolic events during treatment and their median overall survival was significantly shorter than those patients who did not have thrombolic events (9.5 vs. 12.9 months; *p* = 0.03) (Mellema et al., 2014). Much evidence has shown that cancer patients using TCM have a significantly reduced risk of death (Kuo et al., 2018), especially those with acute myeloid leukemia (Tsai et al., 2017), chronic lymphocytic leukemia (Fleischer et al., 2016), advanced breast cancer (Lee et al., 2014), nasopharyngeal cancer (Song et al., 2019), head and neck cancer (Lin et al., 2015), lung cancer (Shen & Wen, 2018), gastric cancer (Hung et al., 2017), liver cancer (Liao et al., 2015), pancreatic cancer (Kuo et al., 2018), or metastatic prostate cancer (Liu et al., 2016). Our data analysis has revealed a highly significant reduction in mortality risk among chemotherapy recipients who used TCM compared with those who did not, with Kaplan-Meier 5-year cumulative incidence mortality rates of 30 and 50%, respectively (Log-rank test, *p* < 0.0001); this mortality benefit with TCM persisted from the end of the second year of follow-up to the end of year 5.

Several limitations surround this study. First, in relation to patient conditions, the LHID2000 data contain details of age, comorbidities, and patients' past medical histories, although as our population was Asian, we could not analyze the data for racial differences. Second, we were limited to ICD-9-CM codes for our analyses, as ICD-10-CM diagnosis codes were not adopted by the NHIRD until 2016. Third, as the LHID2000 cannot provide information on cancer staging at the time of diagnosis, we considered death as a competing risk to minimize the effect of the stage of cancer. We used the Fine and Gray model to estimate the subdistribution hazard ratios (sHRs) of stroke by considering death as a competing risk. Moreover, we applied the Aalen-Johansen estimator to compare Kaplan-Meier analyses with competing risk cumulative incidence curves. Fourth, we did not obtain all necessary data on stroke etiology. The LHID2000 cannot provide data on cigarette smoking and alcohol intake, body weight, height, dietary nutrition and physical activity. However, we were able to consider most of the major vascular risk factors that could reflect background etiology. Furthermore, our Fine and Gray analysis adjusted for medications such as aspirin and the hypercoagulation status of cancer patients, such as coagulopathy (ICD-9-CM code 286.9) and disseminated intravascular coagulopathy (ICD-9-CM code 286.6), to reduce the influence of mediators and confounders on the incidence of stroke. Finally, the CHMs that are commonly used in cancer patients receiving chemotherapy were selected for our analysis by the order of prescription frequency; the accuracy of their purported pharmacological mechanisms or clinical efficacy for reducing the risk of stroke in patients with malignant neoplasms should be evaluated in basic studies or prospective clinical trials.

## Conclusion

In summary, cancer with chemotherapy is a risk factor for stroke. TCM appears to be a safe treatment option that is associated with a reduced risk of stroke and mortality when administered concurrently with chemotherapy in Taiwan. Our findings deserve to be explored in further pharmacological investigations and prospective clinical trials.

## Data Availability

The original contributions presented in the study are included in the article/Supplementary Material, further inquiries can be directed to the corresponding author.

## References

[B1] Adams H. P. Jr. (2019). Cancer and Cerebrovascular Disease. Curr. Neurol. Neurosci. Rep. 19 (10), 73. 10.1007/s11910-019-0985-0 31440841

[B2] Cabioglu M. T. Ergene N. (2005). Electroacupuncture Therapy for Weight Loss Reduces Serum Total Cholesterol, Triglycerides, and LDL Cholesterol Levels in Obese Women. Am. J. Chin. Med. 33 (4), 525–533. 10.1142/S0192415X05003132 16173527

[B3] Cabioglu M. T. Gündoǧan N. Ergene N. (2008). The Efficacy of Electroacupuncture Therapy for Weight Loss Changes Plasma Lipoprotein A, Apolipoprotein A and Apolipoprotein B Levels in Obese Women. Am. J. Chin. Med. 36 (6), 1029–1039. 10.1142/S0192415X08006430 19051333

[B4] Chang C.-C. Lee Y.-C. Lin C.-C. Chang C.-H. Chiu C.-D. Chou L.-W. (2016). Characteristics of Traditional Chinese Medicine Usage in Patients with Stroke in Taiwan: A Nationwide Population-Based Study. J. Ethnopharmacol. 186, 311–321. 10.1016/j.jep.2016.04.018 27090345

[B45] Chen S. C. (2019). Taiwan Herbal Pharmacopeia 3rd Edition English Version. No.488, Sec. 6, Zhongxiao E. Rd., Nangang Dist., Taipei City 115, Taiwan (R.O.C.): Ministry of health and Welfare.

[B5] Choi D. C. Lee J. Y. Moon Y. J. Kim S. W. Oh T. H. Yune T. Y. (2010). Acupuncture-mediated Inhibition of Inflammation Facilitates Significant Functional Recovery after Spinal Cord Injury. Neurobiol. Dis. 39 (3), 272–282. 10.1016/j.nbd.2010.04.003 20382225

[B6] Dardiotis E. Aloizou A.-M. Markoula S. Siokas V. Tsarouhas K. Tzanakakis G. (2019). Cancer-associated Stroke: Pathophysiology, Detection and Management (Review). Int. J. Oncol. 54 (3), 779–796. 10.3892/ijo.2019.4669 30628661 PMC6365034

[B7] Diener H.-C. Hankey G. J. (2020). Primary and Secondary Prevention of Ischemic Stroke and Cerebral Hemorrhage. J. Am. Coll. Cardiol. 75 (15), 1804–1818. 10.1016/j.jacc.2019.12.072 32299593

[B8] Falanga A. (1998). Mechanisms of Hypercoagulation in Malignancy and during Chemotherapy. Pathophysiol. Haemos Thromb. 28 (Suppl. 3), 50–60. 10.1159/000022405 10069762

[B9] Fine J. P. Gray R. J. (1999). A Proportional Hazards Model for the Subdistribution of a Competing Risk. J. Am. Stat. Assoc. 94 (446), 496–509. 10.1080/01621459.1999.10474144

[B10] Flachskampf F. A. Gallasch J. Gefeller O. Gan J. Mao J. Pfahlberg A. B. (2007). Randomized Trial of Acupuncture to Lower Blood Pressure. Circulation 115 (24), 3121–3129. 10.1161/CIRCULATIONAHA.106.661140 17548730

[B11] Fleischer T. Chang T.-T. Chiang J.-H. Hsieh C.-Y. Sun M.-F. Yen H.-R. (2016). Integration of Chinese Herbal Medicine Therapy Improves Survival of Patients with Chronic Lymphocytic Leukemia. Medicine 95 (21), e3788. 10.1097/MD.0000000000003788 27227953 PMC4902377

[B12] Gooley T. A. Leisenring W. Crowley J. Storer B. E. (1999). Estimation of Failure Probabilities in the Presence of Competing Risks: New Representations of Old Estimators. Statist. Med. 18 (6), 695–706. 10.1002/(sici)1097-0258(19990330)18:6<695::aid-sim60>3.0.co;2-o 10204198

[B13] Graus F. Rogers L. R. Posner J. B. (1985). Cerebrovascular Complications in Patients with Cancer. Medicine 64 (1), 16–35. 10.1097/00005792-198501000-00002 3965856

[B14] Grisold W. Oberndorfer S. Struhal W. (2009). Stroke and Cancer: a Review. Acta Neurol. Scand. 119 (1), 1–16. 10.1111/j.1600-0404.2008.01059.x 18616624

[B15] Hung I.-L. Hung Y.-C. Wang L.-Y. Hsu S.-F. Chen H.-J. Tseng Y.-J. (2015). Chinese Herbal Products for Ischemic Stroke. Am. J. Chin. Med. 43 (7), 1365–1379. 10.1142/S0192415X15500779 26477801

[B17] Hung K.-F. Hsu C.-P. Chiang J.-H. Lin H.-J. Kuo Y.-T. Sun M.-F. (2017). Complementary Chinese Herbal Medicine Therapy Improves Survival of Patients with Gastric Cancer in Taiwan: A Nationwide Retrospective Matched-Cohort Study. J. Ethnopharmacol. 199, 168–174. 10.1016/j.jep.2017.02.004 28163114

[B16] Hung Y.-C. Cheng Y.-C. Muo C.-H. Chiu H. E. Liu C.-T. Hu W.-L. (2016). Adjuvant Chinese Herbal Products for Preventing Ischemic Stroke in Patients with Atrial Fibrillation. PLoS One 11 (7, e0159333), e0159333. 10.1371/journal.pone.0159333 27428543 PMC4948896

[B18] Ip F. C.-F. Zhao Y.-M. Chan K.-W. Cheng E. Y.-L. Tong E. P.-S. Chandrashekar O. (2016). Neuroprotective Effect of a Novel Chinese Herbal Decoction on Cultured Neurons and Cerebral Ischemic Rats. BMC Complement. Altern. Med. 16 (1), 437. 10.1186/s12906-016-1417-1 27814708 PMC5097373

[B19] Jang H.-S. Choi J. Shin J. Chung J.-W. Bang O. Y. Kim G.-M. (2019). The Long-Term Effect of Cancer on Incident Stroke: A Nationwide Population-Based Cohort Study in Korea. Front. Neurol. 10, 52. 10.3389/fneur.2019.00052 30804874 PMC6370617

[B20] Kanjanapan Y. Gilbourd D. Pranavan G. (2020). Acute Ischaemic Stroke Following Cisplatin-Based Chemotherapy for Testicular Cancer. BMJ Case Rep. 13 (5), e235005. 10.1136/bcr-2020-235005 PMC7247405 32439748

[B21] Kim D. D. Pica A. M. Durán R. G. Durán W. N. (2006). Acupuncture Reduces Experimental Renovascular Hypertension through Mechanisms Involving Nitric Oxide Synthases. Microcirculation 13 (7), 577–585. 10.1080/10739680600885210 16990216 PMC1618823

[B22] Kim H. T. (2007). Cumulative Incidence in Competing Risks Data and Competing Risks Regression Analysis. Clin. Cancer Res. 13 (2 Pt 1), 559–565. 10.1158/1078-0432.CCR-06-1210 17255278

[B23] Kitano T. Sasaki T. Gon Y. Todo K. Okazaki S. Kitamura T. (2020). The Effect of Chemotherapy on Stroke Risk in Cancer Patients. Thromb. Haemost. 120 (4), 714–723. 10.1055/s-0040-1708484 32289866

[B24] Kuo Y.-T. Chang T.-T. Muo C.-H. Wu M.-Y. Sun M.-F. Yeh C.-C. (2018). Use of Complementary Traditional Chinese Medicines by Adult Cancer Patients in Taiwan: A Nationwide Population-Based Study. Integr. Cancer Ther. 17 (2), 531–541. 10.1177/1534735417716302 28665160 PMC6041896

[B25] Kuo Y.-T. Liao H.-H. Chiang J.-H. Wu M.-Y. Chen B.-C. Chang C.-M. (2018). Complementary Chinese Herbal Medicine Therapy Improves Survival of Patients with Pancreatic Cancer in Taiwan: A Nationwide Population-Based Cohort Study. Integr. Cancer Ther. 17 (2), 411–422. 10.1177/1534735417722224 28774207 PMC6041895

[B26] Lai C.-Y. Chiang J.-H. Lin J.-G. Yen H.-R. Tu C.-H. Chen Y.-H. (2018). Chinese Herbal Medicine Reduced the Risk of Stroke in Patients with Parkinson's Disease: A Population-Based Retrospective Cohort Study from Taiwan. PLoS One 13 (9), e0203473. 10.1371/journal.pone.0203473 30192890 PMC6128574

[B27] Lee Y.-W. Chen T.-L. Shih Y.-R. V. Tsai C.-L. Chang C.-C. Liang H.-H. (2014). Adjunctive Traditional Chinese Medicine Therapy Improves Survival in Patients with Advanced Breast Cancer: a Population-Based Study. Cancer 120 (9), 1338–1344. 10.1002/cncr.28579 24496917

[B28] Li S.-H. Chen W.-H. Tang Y. Rau K.-M. Chen Y.-Y. Huang T.-L. (2006). Incidence of Ischemic Stroke post-chemotherapy: a Retrospective Review of 10,963 Patients. Clin. Neurol. Neurosurg. 108 (2), 150–156. 10.1016/j.clineuro.2005.03.008 16412836

[B29] Li X. Lin J. Han W. Mai W. Wang L. Li Q. (2012). Antioxidant Ability and Mechanism of Rhizoma Atractylodes Macrocephala. Molecules 17 (11), 13457–13472. 10.3390/molecules171113457 23149564 PMC6268131

[B30] Liao C.-C. Lin J.-G. Tsai C.-C. Lane H.-L. Su T.-C. Wang H.-H. (2012). An Investigation of the Use of Traditional Chinese Medicine in Stroke Patients in Taiwan. Evid.-Based Complement. Altern. Med. 2012, 1–8. 10.1155/2012/387164 PMC3530233 23304199

[B31] Liao Y.-H. Lin C.-C. Lai H.-C. Chiang J.-H. Lin J.-G. Li T.-C. (2015). Adjunctive Traditional Chinese Medicine Therapy Improves Survival of Liver Cancer Patients. Liver Int. 35 (12), 2595–2602. 10.1111/liv.12847 25875878

[B32] Lin H.-C. Lin C.-L. Huang W.-Y. Shangkuan W.-C. Kang B.-H. Chu Y.-H. (2015). The Use of Adjunctive Traditional Chinese Medicine Therapy and Survival Outcome in Patients with Head and Neck Cancer: a Nationwide Population-Based Cohort Study. QJM 108 (12), 959–965. 10.1093/qjmed/hcv079 25862772

[B33] Liu J. Wang S. Zhang Y. Fan H.-t. Lin H.-s. (2015). Traditional Chinese Medicine and Cancer: History, Present Situation, and Development. Thorac. Cancer 6 (5), 561–569. 10.1111/1759-7714.12270 26445604 PMC4567000

[B34] Liu J.-M. Lin P.-H. Hsu R.-J. Chang Y.-H. Cheng K.-C. Pang S.-T. (2016). Complementary Traditional Chinese Medicine Therapy Improves Survival in Patients with Metastatic Prostate Cancer. Medicine 95 (31), e4475. 10.1097/MD.0000000000004475 27495088 PMC4979842

[B35] Liu T. Ding Y. Wen A. (2018). Traditional Chinese Medicine for Ischaemic Stroke. Lancet Neurol. 17 (9), 745. 10.1016/S1474-4422(18)30290-4 30129474

[B37] Lyman G. H. Bohlke K. Khorana A. A. Kuderer N. M. Lee A. Y. Arcelus J. I. (2015). Venous Thromboembolism Prophylaxis and Treatment in Patients with Cancer: American Society of Clinical Oncology Clinical Practice Guideline Update 2014. Jco 33 (6), 654–656. 10.1200/JCO.2014.59.7351 PMC4881372 25605844

[B36] Lyman G. H. Khorana A. A. Falanga A. Clarke-Pearson D. Flowers C. Jahanzeb M. (2007). American Society of Clinical Oncology Guideline: Recommendations for Venous Thromboembolism Prophylaxis and Treatment in Patients with Cancer. Jco 25 (34), 5490–5505. 10.1200/JCO.2007.14.1283 17968019

[B38] Mellema W. W. van der Hoek D. Postmus P. E. Smit E. F. (2014). Retrospective Evaluation of Thromboembolic Events in Patients with Non-small Cell Lung Cancer Treated with Platinum-Based Chemotherapy. Lung Cancer 86 (1), 73–77. 10.1016/j.lungcan.2014.07.017 25129368

[B39] Meschia J. F. Bushnell C. Boden-Albala B. Braun L. T. Bravata D. M. Chaturvedi S. (2014). Guidelines for the Primary Prevention of Stroke. Stroke 45 (12), 3754–3832. 10.1161/STR.0000000000000046 25355838 PMC5020564

[B40] Saynak M. Cosar-Alas R. Yurut-Caloglu V. Caloglu M. Kocak Z. Uzal C. (2008). Chemotherapy and Cerebrovascular Disease. J. BUON 13 (1), 31–36. 18404783

[B41] Shen H.-S. Wen S.-H. (2018). Effect of Early Use of Chinese Herbal Products on Mortality Rate in Patients with Lung Cancer. J. Ethnopharmacol. 211, 1–8. 10.1016/j.jep.2017.09.025 28942131

[B42] Shih C.-C. Liao C.-C. Sun M.-F. Su Y.-C. Wen C.-P. Morisky D. E. (2015). A Retrospective Cohort Study Comparing Stroke Recurrence Rate in Ischemic Stroke Patients with and without Acupuncture Treatment. Medicine 94 (39), e1572. 10.1097/MD.0000000000001572 26426630 PMC4616848

[B43] Song Y. C. Hung K. F. Liang K. L. Chiang J. H. Huang H. C. Lee H. J. (2019). Adjunctive Chinese Herbal Medicine Therapy for Nasopharyngeal Carcinoma: Clinical Evidence and Experimental Validation. Head Neck 41 (9), 2860–2872. 10.1002/hed.25766 30985039

[B44] Taccone F. S. Jeangette S. M. Blecic S. A. (2008). First-ever Stroke as Initial Presentation of Systemic Cancer. J. Stroke Cerebrovasc. Dis. 17 (4), 169–174. 10.1016/j.jstrokecerebrovasdis.2008.01.007 18589335

[B46] Tsai M.-Y. Hu W.-L. Chiang J.-H. Huang Y.-C. Chen S.-Y. Hung Y.-C. (2017). Improved Medical Expenditure and Survival with Integration of Traditional Chinese Medicine Treatment in Patients with Heart Failure: A Nationwide Population-Based Cohort Study. Oncotarget 8 (52), 90465–90476. 10.18632/oncotarget.20063 29163845 PMC5685766

[B47] Tully C. M. Apolo A. B. Zabor E. C. Regazzi A. M. Ostrovnaya I. Furberg H. F. (2016). The High Incidence of Vascular Thromboembolic Events in Patients with Metastatic or Unresectable Urothelial Cancer Treated with Platinum Chemotherapy Agents. Cancer 122 (5), 712–721. 10.1002/cncr.29801 26618338 PMC4990408

[B48] Xiang Y. Guo Z. Zhu P. Chen J. Huang Y. (2019). Traditional Chinese Medicine as a Cancer Treatment: Modern Perspectives of Ancient but Advanced Science. Cancer Med. 8 (5), 1958–1975. 10.1002/cam4.2108 30945475 PMC6536969

[B49] Yang S.-H. Yu C.-L. Chen H.-Y. Lin Y.-H. (2013). A Commonly Used Chinese Herbal Formula, Shu-Jing-Hwo-Shiee-Tang, Potentiates Anticoagulant Activity of Warfarin in a Rabbit Model. Molecules 18 (10), 11712–11723. 10.3390/molecules181011712 24071980 PMC6270155

[B50] Yeh M.-L. Chiu W.-L. Wang Y.-J. Lo C. (2017). An Investigation of the Use of Traditional Chinese Medicine and Complementary and Alternative Medicine in Stroke Patients. Holist. Nurs. Pract. 31 (6), 400–407. 10.1097/HNP.0000000000000238 29028779

