# Supplementary material for: Traditional Chinese Medicine Reduces the Incidence of Chemotherapy-Induced Stroke: A Five-Year Nationwide Population-Based Cohort Study From Taiwan
Source: Front Pharmacol. 2021 May 26;12:614606. doi: 10.3389/fphar.2021.614606 (PMC8187954; doi:10.3389/fphar.2021.614606)
Supplement: Supplementary file 1 [file DataSheet1.docx]

Supplementary material

For

Traditional Chinese Medicine Reduces the Incidence of Chemotherapy-Induced Stroke: a Five-year Nationwide Population-based Cohort Study From Taiwan

Chien-Chen Huang, Yu-Cih Yang, Iona MacDonald, Ching-Yuan Lai, Cheng-Hao Tu, Yi-Hung Chen

Supplementary Tables S1–10

**Supplementary Table S1.** Baseline characteristics for CT users and non-CT users (controls)

|  | CT users (n=3,054) | Non-CT users (n=3,054) | p-value |
| --- | --- | --- | --- |
| **Gender (n, %)** |  |  | >0.99^**^ |
| Female | 1,238 (40.5) | 1,238 (40.5) |  |
| Male | 1,816 (59.5) | 1,816 (59.5) |  |
| **Age, years (n, %)** |  |  | 0.73^**^ |
| 20–29 | 62 (2.03) | 65 (2.13) |  |
| 30–39 | 216 (7.07) | 205 (6.71) |  |
| 40–49 | 598 (19.6) | 620 (20.3) |  |
| 50–59 | 682 (22.3) | 638 (20.9) |  |
| 60–69 | 690 (22.6) | 718 (23.5) |  |
| ≥70 | 806 (26.4) | 808 (26.4) |  |
| **Mean (SD)** | 59.08 (14.2) | 59.04 (14.2) | 0.99^*^ |
| **Cancer-related therapy (n, %)** |  |  |  |
| **Surgery** | 964 (31.6) | 964 (31.6) | >0.99^**^ |
| **Radiotherapy** | 387 (12.7) | 387 (12.7) | >0.99^**^ |
| **Medication (n, %)** |  |  |  |
| Immune therapy | 0 (0) | 0 (0) | -- |
| Targeted therapy | 12 (0.39) | 12 (0.39) | >0.99^**^ |
| Hormonal therapy | 86 (2.82) | 86 (2.82) | >0.99^**^ |
| **Stroke-related comorbidities (n, %)** |  |  |  |
| Hyperlipidemia | 90 (2.95) | 90 (2.95) | >0.99^**^ |
| Diabetes mellitus | 120 (3.93) | 120 (3.93) | >0.99^**^ |
| Hypertensive disease | 88 (2.88) | 88 (2.88) | >0.99^**^ |
| Coronary heart disease | 41 (1.34) | 41 (1.34) | >0.99^**^ |
| Acute pulmonary heart disease | 0 (0) | 0 (0) | -- |
| Cardiac dysrhythmia | 13 (0.43) | 13 (0.43) | >0.99^**^ |
| Congestive heart failure | 30 (0.98) | 30 (0.98) | >0.99^**^ |
| Atherosclerosis | 0 (0) | 0 (0) | >0.99^**^ |
| Peripheral vascular disease | 2 (0.07) | 2 (0.07) | >0.99^**^ |
| Other venous embolism and thrombosis | 1 (0.03) | 1 (0.03) | >0.99^**^ |
| **Mean (SD) follow-up for stroke (years)** | 2.19 (1.99) | 3.64 (1.83) | <0.0001^*^ |
| **Mean (SD) follow-up for death (years)** | 2.24 (2.01) | 3.81 (1.77) | <0.0001^*^ |
| Abbreviations: CT = chemotherapy; SD = standard deviation.  -- Unable to calculate because there were either too few or no events.  *Two-sample *t*-test; **Chi-square test. | | | |

**Supplementary Table S2.** Cancer types among chemotherapy users and non-chemotherapy users (controls) at 5 years of follow-up

| **Cancer type**  **(ICD-9-CM)^a^** | **CT users (n=3,054)**  **n, %** | **Non-CT users (n=3,054) n, %** | ***p*-value** |
| --- | --- | --- | --- |
| 140-146,148-149 | 199 (6.52) | 172 (5.63) | 0.14 |
| 147 | 81 (2.65) | 88 (2.88) | 0.58 |
| 150 | 55 (1.80) | 47 (1.54) | 0.42 |
| 151 | 255 (8.35) | 92 (0.31) | <0.0001 |
| 153, 154 | 610 (19.9) | 371 (12.1) | <0.0001 |
| 155 | 562 (18.4) | 532 (17.4) | 0.31 |
| 157 | 68 (2.23) | 58 (1.90) | 0.36 |
| 160 | 7 (0.23) | 7 (0.23) | >0.99 |
| 161 | 14 (0.46) | 30 (0.98) | 0.01 |
| 162 | 333 (10.9) | 319 (10.4) | 0.56 |
| 171 | 16 (0.52) | 26 (0.85) | 0.12 |
| 172 | 4 (0.13) | 12 (0.39) | 0.04 |
| 173 | 30 (0.98) | 68 (2.23) | <0.0001 |
| 174 | 222 (7.27) | 154 (5.04) | 0.0003 |
| 180 | 61 (2.00) | 154 (5.04) | <0.0001 |
| 182 | 18 (0.59) | 32 (1.05) | 0.04 |
| 183 | 73 (2.39) | 43 (1.41) | 0.004 |
| 184 | 2 (0.07) | 3 (0.10) | 0.65 |
| 185 | 19 (0.62) | 204 (6.68) | <0.0001 |
| 188 | 83 (2.72) | 106 (3.47) | 0.08 |
| 191 | 21 (0.69) | 51 (1.67) | 0.0004 |
| 192 | 1 (0.03) | 9 (0.29) | 0.01 |
| 193 | 7 (0.23) | 59 (1.93) | <0.0001 |
| 200-208 | 169 (5.53) | 57 (1.87) | <0.0001 |
| Others (152,156, 158,159, 163-165, 170, 175-176,179, 181, 186-187,189, 190, 192, 194-199, 209) | 536 (17.5) | 423 (13.8) | <0.0001 |
| Abbreviation: CT = chemotherapy.  ^a^ICD-9-CM codes: 140-146, Malignant neoplasm of lip, tongue, major salivary glands, gum, floor of mouth, other and unspecified parts of mouth, and oropharynx; 147, Malignant neoplasm of nasopharynx; 148-149, Malignant neoplasm of hypopharynx, and other and ill-defined sites within the lip, oral cavity, and pharynx; 150, Malignant neoplasm of esophagus; 151, Malignant neoplasm of stomach; 153, Malignant neoplasm of colon; 154, Malignant neoplasm of rectum, rectosigmoid junction, and anus; 155, Malignant neoplasm of liver and intrahepatic bile ducts; 162, Malignant neoplasm of trachea, bronchus, and lung; 173, Other malignant neoplasm of skin; 174, Malignant neoplasm of female breast; 180, Malignant neoplasm of cervix uteri; 182, Malignant neoplasm of body of uterus; 183, Malignant neoplasm of ovary and other uterine adnexa; 185, Malignant neoplasm of prostate; 188, Malignant neoplasm of bladder; 191, Malignant neoplasm of brain. 192 Malignant neoplasm of other and unspecified parts of nervous system; 193, Malignant neoplasm of thyroid gland; 200, Lymphosarcoma and reticulosarcoma and other specified malignant tumors of lymphatic tissue; 201, Hodgkin's disease; 202, Other malignant neoplasms of lymphoid and histiocytic tissue; 203, Multiple myeloma and immunoproliferative neoplasms; 204, Lymphoid leukemia; 205, Myeloid leukemia; 206, Monocytic leukemia; 207, Other specified leukemia; 208, Leukemia of unspecified cell type. | | | |

**Supplementary Table S3.** Cancer types among chemotherapy users with TCM or without TCM (controls), at 5 years of follow-up

| **Cancer type**  **(ICD-9-CM)^a^** | **TCM users (n=593)**  **n, %** | **Non-TCM users**  **(n=593) n, %** | ***p-*value** |
| --- | --- | --- | --- |
| 140-146, 148-149 | 33 (5.56) | 61 (10.3) | 0.002 |
| 147 | 24 (4.05) | 18 (3.04) | 0.34 |
| 150 | 21 (3.54) | 34 (5.73) | 0.07 |
| 151 | 41 (6.91) | 47 (7.93) | 0.50 |
| 153, 154 | 111 (18.7) | 86 (14.5) | 0.05 |
| 155 | 122 (20.6) | 86 (14.5) | 0.006 |
| 157 | 13 (2.19) | 16 (2.70) | 0.57 |
| 160 | 2 (0.34) | 2 (0.34) | >0.99 |
| 161 | 1 (0.17) | 4 (0.67) | 0.17 |
| 162 | 50 (8.43) | 82 (13.8) | 0.003 |
| 171 | 4 (0.67) | 3 (0.51) | 0.70 |
| 172 | 4 (0.67) | 2 (0.34) | 0.41 |
| 173 | 3 (0.51) | 4 (0.67) | 0.70 |
| 174 | 51 (8.60) | 30 (5.06) | 0.01 |
| 180 | 9 (1.52) | 12 (2.02) | 0.50 |
| 182 | 4 (0.67) | 4 (0.67) | >0.99 |
| 183 | 8 (1.35) | 14 (2.36) | 0.19 |
| 184 | 0 | 0 | -- |
| 185 | 3 (0.51) | 5 (0.84) | 0.47 |
| 188 | 17 (2.87) | 7 (1.18) | 0.03 |
| 191 | 1 (0.17) | 4 (0.67) | 0.17 |
| 192 | 1 (0.17) | 0 | 0.31 |
| 193 | 1 (0.17) | 0 | 0.31 |
| 200-208 | 31 (5.23) | 47 (7.93) | 0.06 |
| Others  (152,156, 158,159, 163-165, 170, 175-176,179, 181, 186-187,189, 190, 192, 194-199, 209) | 102 (17.2) | 115 (19.4) | 0.32 |
| Abbreviation: TCM = traditional Chinese medicine.  ^a^ICD-9-CM codes: 140-146, Malignant neoplasm of lip, tongue, major salivary glands, gum, floor of mouth, other and unspecified parts of mouth, and oropharynx; 147, Malignant neoplasm of nasopharynx; 148-149, Malignant neoplasm of hypopharynx, and other and ill-defined sites within the lip, oral cavity, and pharynx; 150, Malignant neoplasm of esophagus; 151, Malignant neoplasm of stomach; 153, Malignant neoplasm of colon; 154, Malignant neoplasm of rectum, rectosigmoid junction, and anus; 155, Malignant neoplasm of liver and intrahepatic bile ducts; 162, Malignant neoplasm of trachea, bronchus, and lung; 173, Other malignant neoplasm of skin; 174, Malignant neoplasm of female breast; 180, Malignant neoplasm of cervix uteri; 182, Malignant neoplasm of body of uterus; 183, Malignant neoplasm of ovary and other uterine adnexa; 185, Malignant neoplasm of prostate; 188, Malignant neoplasm of bladder; 191, Malignant neoplasm of brain. 192 Malignant neoplasm of other and unspecified parts of nervous system; 193, Malignant neoplasm of thyroid gland; 200, Lymphosarcoma and reticulosarcoma and other specified malignant tumors of lymphatic tissue; 201, Hodgkin's disease; 202, Other malignant neoplasms of lymphoid and histiocytic tissue; 203, Multiple myeloma and immunoproliferative neoplasms; 204, Lymphoid leukemia; 205, Myeloid leukemia; 206, Monocytic leukemia; 207, Other specified leukemia; 208, Leukemia of unspecified cell type. | | | |

**Supplementary Table S4.** Incidence rates of stroke among chemotherapy and non-chemotherapy users (controls) between Jan 1, 2000, and Dec 31, 2006, over the 5 years of follow-up

|  | **At 1 year** | | **At 2 years** | | **At 3 years** | | **At 4 years** | | **At 5 years** | |
| --- | --- | --- | --- | --- | --- | --- | --- | --- | --- | --- |
|  | **CT users (n=1,364)** | **Controls (n=536)** | **CT users (n=1,771)** | **Controls (n=798)** | **CT users (n=1,987)** | **Controls (n=988)** | **CT users (n=2,130)** | **Controls (n=1,149)** | **CT users (n=3,054)** | **Controls (n=3,054)** |
| Numbers of patients with stroke | 87 | 51 | 140 | 82 | 187 | 102 | 227 | 115 | 252 | 122 |
| Incidence per 100 PY | 279 | 523 | 660 | 1,098 | 1,115 | 1,625 | 1,669 | 2119 | 11,120 | 6,693 |
| Incidence rates | 31.1 | 9.75 | 21.2 | 7.46 | 16.7 | 6.27 | 13.6 | 5.42 | 2.26 | 1.82 |
| Crude sHR  (95% CI) | 2.84  (2.00–4.04)*** | 1 | 2.75  (2.09–3.62)*** | 1 | 2.53  (1.99–3.23)*** | 1 | 2.32  (1.85–2.91)*** | 1 | 1.31  (1.06–1.63)** | 1 |
| Adjusted sHR^††^  (95% CI) | 2.21  (1.52–3.20)*** | 1 | 2.32  (1.74–3.09)*** | 1 | 2.08  (1.61–2.69)*** | 1 | 1.94  (1.53–2.46)*** | 1 | 1.15  (0.92–1.44) | 1 |
| Abbreviations: CT = chemotherapy; PY = person-years; sHR = subdistribution hazard ratio; CI = confidence interval.  ^††^ The model was adjusted by gender, age, index year, cancer-related therapy and stroke-related comorbidities, aspirin use, coagulopathy and disseminated intravascular coagulopathy. All-cause mortality was considered to be a competing risk event.  **p<0.01; ***p<0.001. | | | | | | | | | | |

**Supplementary Table S5.** Incidence rates of stroke among TCM users and non-TCM users (controls) between Jan 1, 2000 and Dec 31, 2006, over the 5 years of follow-up, with three layers of stratification

|  | < **1 year** | | **≧1 year and <2 years** | | **≧2 years and ≦5 years** | |
| --- | --- | --- | --- | --- | --- | --- |
|  | **TCM users (n=273)** | **Controls (n=435)** | **TCM users (n=72)** | **Controls (n=68)** | **TCM users (n=248)** | **Controls (n=90)** |
| Numbers of patients with stroke | 10 | 21 | 5 | 3 | 11 | 4 |
| Incidence per 100 PY | 111 | 130 | 106 | 97 | 1189 | 376 |
| Incidence rates | 9.00 | 16.1 | 4.71 | 3.09 | 0.92 | 1.06 |
| Crude sHR  (95% CI) | 0.56  (0.26–1.20) | 1 | 1.14  (0.26–4.89) | 1 | 0.91  (0.29–2.86) | 1 |
| Adjusted sHR^††^  (95% CI) | 0.68  (0.31–1.50) | 1 | 1.20  (0.24–5.97) | 1 | 0.73  (0.53–0.97)* | 1 |
| Abbreviations: CT = chemotherapy; PY = person-years; sHR = subdistribution hazard ratio; CI = confidence interval.  †† The model was adjusted by gender, age, index year, cancer-related therapy and stroke-related comorbidities, aspirin use, coagulopathy and disseminated intravascular coagulopathy. All-cause mortality was considered to be a competing risk event.  *p<0.05. | | | | | | |

**Supplementary Table S6.** Incidence rates of stroke among TCM users and non-TCM users (controls) between Jan 1, 2000 and Dec 31, 2006, over the 5 years of follow-up

|  | **At 1 year** | | **At 2 years** | | **At 3 years** | | **At 4 years** | | **At 5 years** | |
| --- | --- | --- | --- | --- | --- | --- | --- | --- | --- | --- |
|  | **TCM users (n=273)** | **Controls (n=435)** | **TCM users (n=345)** | **Controls (n=503)** | **TCM users (n=387)** | **Controls (n=526)** | **TCM users (n=411)** | **Controls (n=533)** | **TCM users (n=593)** | **Controls (n=593)** |
| Numbers of patients with stroke | 10 | 21 | 15 | 24 | 19 | 27 | 26 | 28 | 26 | 28 |
| Incidence per 10 PY | 111 | 130 | 217 | 227 | 320 | 282 | 405 | 306 | 1306 | 604 |
| Incidence rates | 0.9 | 1.61 | 0.69 | 1.05 | 0.59 | 0.95 | 0.64 | 0.91 | 0.19 | 0.46 |
| Crude sHR  (95% CI) | 0.56  (0.26–1.20) | 1 | 0.62  (0.32–1.19) | 1 | 0.57  (0.31–1.05) | 1 | 0.57  (0.32–1.00)* | 1 | 0.49  (0.28–0.85)* | 1 |
| Adjusted sHR^††^  (95% CI) | 0.68  (0.31–1.50) | 1 | 0.68  (0.32–1.28) | 1 | 0.58  (0.30–1.09) | 1 | 0.53  (0.29–0.97)* | 1 | 0.45  (0.26–0.79)*** | 1 |
| Abbreviations: TCM = traditional Chinese medicine; PY = person-years; sHR = subdistribution hazard ratio; CI = confidence interval.  ^††^ The model was adjusted by gender, age, index year, cancer-related therapy and stroke-related comorbidities, aspirin use, coagulopathy and disseminated intravascular coagulopathy. All-cause mortality was considered to be a competing risk event.  *p<0.05; ***p<0.001. | | | | | | | | | | |

**Supplementary Table S7.** Risk of stroke among chemotherapy users with TCM or without TCM (controls), at 5 years of follow-up

|  | **Hemorrhagic stroke** | | **Ischemic stroke** | | **Transient cerebral ischemia** | | **Ill-defined cerebral vascular disease** | |
| --- | --- | --- | --- | --- | --- | --- | --- | --- |
|  | **TCM users (n=26)** | **Controls (n=28)** | **TCM users (n=26)** | **Controls (n=28)** | **TCM users (n=26)** | **Controls (n=28)** | **TCM users (n=26)** | **Controls (n=28)** |
| Numbers of patients with stroke | 6 | 11 | 9 | 6 | 3 | 2 | 8 | 9 |
| Percentages (%) | 23.1 | 39.2 | 34.6 | 21.4 | 11.5 | 7.14 | 30.8 | 32.1 |
| Crude sHR  (95% CI) | 0.34  (0.09–1.26) | 1 | 0.42  (0.15–1.15) | 1 | 0.13  (0.01–1.50) | 1 | 0.31  (0.11–0.84)* | 1 |
| Adjusted sHR^††^  (95% CI) | 0.34  (0.06–1.89) | 1 | 0.11  (0.02–0.62)* | 1 | -- | -- | 0.20  (0.04–1.01) | 1 |
| Abbreviations: TCM = traditional Chinese medicine; sHR = subdistribution hazard ratio; CI = confidence interval.  †† The model was adjusted by gender, age, index year, cancer-related therapy and stroke-related comorbidities, aspirin-use, coagulopathy and disseminated intravascular coagulopathy. All-cause mortality was considered to be a competing risk event.  -- Unable to calculate because there were either too few or no events.  *p<0.05. | | | | | | | | |

**Supplementary Table S8.** The 10 most commonly prescribed Chinese formulas for cancer patients with or without stroke who used chemotherapy between Jan 1, 2000 and Dec 31, 2006, at 5 years of follow-up

| **Traditional Chinese formula name** | **Numbers of prescriptions** | **Average daily doses (g)** | **Average duration of prescription (days)** | **Effects** |
| --- | --- | --- | --- | --- |
| **TCM users without stroke (n=560)** | | | | |
| Xiang-Sha-Liu-Jun-Zi-Tang (XSLJZT) | 355 | 4.5 | 9.9 | Fortify the spleen and nourish the stomach |
| Ban-Xia-Xie-Xin-Tang (BXXXT) | 247 | 7.0 | 7.1 | Harmonize the stomach to downbear counterflow |
| Xiao-Chai-Hu-Tang (XCHT) | 237 | 6.2 | 8 | Harmonize and release the lesser yang |
| Gan-Lu-Yin (GLY) | 226 | 12.2 | 7.7 | Nourish yin and clear dampness-heat |
| Bu-Zhong-Yi-Qi-Tang (BZYQT) | 216 | 4.4 | 10.9 | Tonify the middle and replenish qi, harmonize and tonify the spleen and stomach |
| Suan-Zao-Ren-Tang (SZRT) | 213 | 8.7 | 7.8 | Nourish blood and tranquilize the mind, clear heat and relax the mind |
| Shao-Yao-Gan-Cao-Tang (SYGCT) | 210 | 17.8 | 7.5 | Relax tesion to relieve pain |
| Jia-Wei-Xiao-Yao-San (JWXYS) | 176 | 8.5 | 9.6 | Soothe the liver and release depression, clear heat to cool the blood |
| Shu-Jing-Huo-Xie-Tang (SJHXT) | 174 | 29.7 | 6.9 | Soothe menstruation, activate blood and dispel wind |
| Ma-Zi-Ren-Wan (MZRW) | 173 | 2.8 | 7.8 | Nourish the intestine and discharge heat, promote circulation of Qi and relax the bowels |
| **TCM users with stroke (n=26)** | | | | |
| Ping-Wei-San (PWS) | 37 | 48.3 | 5.9 | Dry dampness to fortify the spleen, regulate qi and harmonize the middle |
| Jing-Fang-Bai-Du-San (JFBDS) | 30 | 5.4 | 6.1 | Promote sweating to release the exterior, disperse wind and dispel dampness |
| Qing-Xin-Li-Ge-Tang (QXLGT) | 28 | 5.5 | 6 | Clear heat and detoxicate |
| Huo-Xiang-Zheng-Qi-San (HXZQS) | 25 | 4.5 | 6.2 | Release the exterior and resolve dampness, regulate qi and harmonize the middle |
| Ban-Xia-Xie-Xin-Tang (BXXXT) | 24 | 90.7 | 6.5 | Harmonize the stomach to downbear counterflow |
| Ma-Xing-Gan-Shi-Tang (MXGST) | 24 | 3 | 6 | Diffusion with pungent-cool, clear the lung to calm panting |
| Xiang-Sha-Liu-Jun-Zi-Tang (XSLJZT) | 23 | 4.4 | 7.1 | Fortify the spleen and nourish the stomach |
| Zhi-Bo-Di-Huang-Wan (ZBDHW) | 22 | 3.3 | 6.8 | Nourish yin to downbear fire |
| Xiao-Chai-Hu-Tang (XCHT) | 21 | 4.2 | 5.6 | Harmonize and release the lesser yang |
| Tiao-Wei-Cheng-Qi-Tang (TWCQT) | 21 | 3.9 | 6.1 | Soften hardness to relax the bowels, harmonize the stomach and discharge heat |

**Supplementary Table S9.** The 10 most commonly prescribed single herbs for cancer patients with or without stroke who used chemotherapy between Jan 1, 2000 and Dec 31, 2006, at 5 years of follow-up

| **Traditional Chinese herbs**  **Pinyin name**  **(official name)** | **Numbers of prescriptions** | **Average daily doses (g)** | **Average duration of prescription (days)** | **Usage** |
| --- | --- | --- | --- | --- |
| **TCM users without stroke (n=560)** | | | | |
| Dan Shen  (*Salvia miltiorrhiza Bunge*) | 478 | 2.5 | 10.6 | Blood-activating and stasis-dispelling medicinal |
| Bai Hua She She Cao  (*Scleromitrion diffusum (Willd.) R. J. Wang*) | 390 | 1.3 | 10.6 | Heat-clearing and detoxcating medicinal |
| Da Huang  (*Rheum palmatum L.*)  *(Rheum tanguticum Maxim. ex Balf.)*  *(Rheum officinale Baill.)* | 346 | 0.9 | 8.7 | Offensive purgative medicinal |
| Yan Hu Suo  (*Corydalis yanhusuo W.t. Wang*) | 255 | 1.2 | 9.3 | Blood-activating and stasis-dispelling medicinal |
| Ban Zhi Lian  (*Scutellariae Barbata D. Don*) | 246 | 1.3 | 13.1 | Heat-clearing and detoxcating medicinal |
| Jhe Bei Mu  (*Fritillariae Thunbergii Miq.*) | 244 | 5.1 | 8.5 | Heat clearing and phlegm-resolving medicinal |
| Huang Qin  (*Scutellaria baicalensis Georgi*) | 225 | 5.6 | 7.9 | Heat-clearing and dampness-drying medicinal |
| Gan Cao  *(Glycyrrhiza uralensis Fisch.)*  *(Glycyrrhiza glabra L.)*  (*Glycyrrhiza inflata Batalin*) | 219 | 1.4 | 7.7 | Qi-tonifying medicinal |
| Gua Lou Gen  (*Trichosanthes rosthornii Harms*) | 195 | 1.1 | 7.7 | Heat-clearing and fire-purging medicinal |
| Mai Men Dong  (*Ophiopogon japonicus (Thunb.) Ker-Gawl*) | 195 | 2.0 | 8.7 | Yin-tonifying medicinal |
| **TCM users with stroke (n=26)** | | | | |
| Bai Hua She She Cao  *(Scleromitrion diffusum (Willd.) R. J.)* | 28 | 1 | 6.5 | Heat-clearing and detoxcating medicinal |
| Hai Piao Siao  *(Sepiella maindroni)*  *(Sepia esculenta)* | 27 | 1 | 5.5 | Astringent medicinal |
| Jie Geng  *(Platycodon grandiflorus (Jacq.) A.DC.)* | 21 | 1.2 | 6.6 | Heat-phlegm clearing and resolving medicinal |
| Ban Jhih Lian  *(Scutellariae Barbatae D. Don)* | 19 | 1.1 | 7.2 | Heat-clearing and detoxcating medicinal |
| Yan Hu Suo  (*Corydalis yanhusuo W.t. Wang*) | 19 | 1.9 | 5.7 | Blood-activating and stasis-dispelling medicinal |
| Yin Chen  *(Artemisia capillaris Thunb.)* | 18 | 1.1 | 7 | Dampness-draining diuretic medicinal |
| Jhe Bei Mu  (*Fritillariae Thunbergii Miq.*) | 18 | 40 | 7.4 | Heat-clearing and phlegm-resolving medicinal |
| Bai Ji  *(Bletilla striata (Thunb.) Rchb.f.)* | 17 | 1.1 | 6.2 | Hemostatic medicinal |
| Suan Zao Ren  *(Ziziphus jujuba Mill.)* | 17 | 1.1 | 6.8 | Heart-nourishing tranquilizing medicinal |
| Fu Shen  *(Poria cocos (Schwein.) F.A.Wolf)* | 16 | 1.1 | 6.8 | Heart-nourishing tranquilizing medicinal |

**Supplementary Table S10.** Incidence rates of mortality among TCM users and non-TCM users (controls) between Jan 1, 2000 and Dec 31, 2006, over the 5 years of follow-up

|  | **At 1 year** | | **At 2 years** | | **At 3 years** | | **At 4 years** | | **At 5 years** | |
| --- | --- | --- | --- | --- | --- | --- | --- | --- | --- | --- |
|  | **TCM users (n=267)** | **Controls (n=424)** | **TCM users (n=337)** | **Controls (n=494)** | **TCM users (n=378)** | **Controls (n=519)** | **TCM users (n=402)** | **Controls (n=526)** | **TCM users (n=593)** | **Controls (n=593)** |
| Numbers of deaths | 101 | 141 | 129 | 167 | 146 | 171 | 154 | 173 | 159 | 178 |
| Incidence per 100 PY | 108 | 130 | 210 | 229 | 312 | 289 | 396 | 313 | 1341 | 643 |
| Incidence rates | 9.35 | 10.8 | 6.14 | 7.29 | 4.67 | 5.91 | 3.88 | 5.52 | 1.18 | 2.76 |
| Crude HR  (95% CI) | 0.83  (0.64–1.07) | 1 | 0.80  (0.64–1.01) | 1 | 0.78  (0.63–0.98)* | 1 | 0.71  (0.57–0.89)** | 1 | 0.54  (0.43–0.67)*** | 1 |
| Adjusted HR^††^  (95% CI) | 0.78  (0.60–1.20) | 1 | 0.78  (0.61–0.98)* | 1 | 0.76  (0.61–0.96)* | 1 | 0.71  (0.57–0.89)** | 1 | 0.55  (0.44–0.68)*** | 1 |
| Abbreviations: TCM = traditional Chinese medicine; PY = person-years; HR = hazard ratio; CI = confidence interval.  ^††^ The model was adjusted by gender, age, index year, cancer-related therapy and stroke-related comorbidities, aspirin-use, coagulopathy and disseminated intravascular coagulopathy. *p<0.05; **p<0.01; ***p<0.001. | | | | | | | | | | |
